# Trade-off between double cleavage-stage embryos transfer and single blastocyst-stage embryo transfer in patients with few good quality embryos in antagonist cycles: a retrospective study using a propensity score matching analysis

**DOI:** 10.1186/s12884-024-06537-5

**Published:** 2024-05-03

**Authors:** Yan Han, Xing Deng, Jiali Cai, Wei Peng, Chaoqun Duan, Kezhen Huang

**Affiliations:** 1 The Assisted Reproduction Department, Yichun Maternal and Child Health Hospital, Yichun, China; 2 Reproductive Medicine Centre, Affifiliated Chenggong Hospital of Xiamen University, Xiamen, China; 3 School of Medicine, Xiamen University, Xiamen, China

**Keywords:** Assisted Reproductive Technology(ART), Embryo transfer(ET), In Vitro fertilization(IVF), Double cleavage-stage embryo transfer(DET), Single blastocyst transfer(SBT), Antagonist

## Abstract

**Objective:**

This study aimed to compare the per OPU clinical outcomes for transfer of Day 3 double cleavage-stage embryos (DET) and Day 5 single blastocyst-stage (SBT) in patients with five or fewer good quality embryos on day 3 per occyte pick-up cycle (OPU) in antagonist cycles with consideration of blastocyst formation failure.

**Methods:**

This was a retrospective, observational cohort study of 2,116 cases of OPU treated with antagonist protocol in the affiliated Chenggong Hospital of Xiamen University between January 2013 and December 2020. DET was performed in 1,811cycles and SBT was performed in 305 cycles. The DET group was matched to the SBT group by propensity score (PS) matching according to multiple maternal baseline covariates. After PS matching, there were 303 ET cycles in each group. The primary outcomes were the cumulative live birth rate (CLBR), cumulative multiple pregnancy rate(CMPR)per OPU and the number of ET to achieve live birth per OPU. Secondary outcomes were the percentage of clinical pregnancy(CPR), live birth rate(LBR), multiple pregnancy rate(MPR).

**Results:**

Following PS mating, the CLBR was slightly higher (48.8% versus 40.3% ; *P* = 0.041) and the CMPR was significantly higher in the DET group compared to SBT group(44.2% versus 7.9%, *P* < 0.001). The CPR, LBR and MPR per fresh transfer were higher in DET group compared to SBT group(50.2% versus 28.7%; 41.3% versus 21.5%;29.6% versus 0%, *P* < 0.001). The number of ET to achieve live birth per OPU in SBT group was obiviously more than in DET group(1.48 ± 0.578 versus 1.22 ± 0.557 ,*P* < 0.001).

**Conclusion:**

With a marginal difference cumulative live birth rate, the lower live birth rate per fresh transfer and higher number of ET per OPU in the SBT group suggested that it might take longer time to achieve a live birth with single blastocyst strategy. A trade-off decision should be made between efficiency and safety.

**Supplementary Information:**

The online version contains supplementary material available at 10.1186/s12884-024-06537-5.

## Introduction

Historically, the practice of assisted reproductive technology (ART) was followed by the transfer of multiple embryos for pursuing higher pregnancy rates [1], but with an increased risk of multiple gestations and a series of neonatal and maternal complications. As the focus is on increasing live birth rates whilst improving the safety of ART and reducing obstetric risks, single embryo transfer (SET) has been advocated as an effective strategy [1–4].

The use of techniques such as extended embryo culture until the blastocyst stage enables a better selection of embryos with a superior developmental capacity and consequently a higher implantation potential [5–8]. It is supposed that blastocyst-stage embryo transfer is a more physiologically appropriate time as it more closely mimics the time of natural implantation and may increase the pregnancy rate and live-birth rate per embryo transferred [9, 10]. Therefore, single blastocyst transfer (SBT) has been applied with an increasing amount in IVF in past years [11]. However, the risk of failed blastocyst formation which results in a reduction in the availability of embryos and even cancellation of the treatment cycle remains a significant consideration [12, 13].

The number and quality of day 3 embryos are important determinants for having viable blastocysts to transfer [14]. There is clear evidence favoring blastocyst transfer versus cleavage-stage embryo transfer in good prognosis patients [15]. However, whether the strategy benefits the patients with a low yield of available embryos remains controversial. Some retrospective studies have demonstrated that extended culture of embryos does not alter implantation potential and the ongoing pregnancy rate when fewer than three embryos are available [16, 19]. Conversely, other authors maintain that extended culture in vitro to the blastocyst stage potentially cancels an embryo transfer that would have resulted in a live birth [17–19]. Xiao et al. evaluated this issue in women with only one embryo available on D3, in whom pregnancy rates were higher when the embryo was transferred on D3 than on D5/6 [20]. As a result, the application of blastocyst transfer in patients with few embryos available on day 3 is still no international guideline or consensus.

Although the ART technique has been improved over decades, the non-physiological environment during extended culture is still considered to be suboptimal and may hamper the blastocyst formation and subsequent development [21, 22]. In a specific case, in vitro arrested embryos that could not achieve blastocyst stage resulted in live birth following multiple cleavage stage embryo transfer [23]. The report suggested that multiple cleavage stage embryo transfer might overcome the situation where extended culture might fail, with the cost of multiple pregnancies. However, study regarding the trade-off between multiple cleavage stage embryo transfer and single blastocyst is still limited.

Therefore, the purpose of this study was to investigate clinical outcomes between single blastocyst transfer and double cleavage-stage embryo transfer in patients with few good embryos. We hope to provide a reference for a more appropriate strategy for embryo transfer.

## Methods

### Study subjects

Institutional review board approval for this retrospective study was obtained from the ethical committee of the Xiamen University Affiliated Chenggong Hospital. Data from 2,116 cycles treated with an antagonistic IVF cycle in the affiliated Chenggong Hospital of Xiamen University between January 2013 and December 2020 were accessed for potential inclusion. The inclusion criteria were the patients receiving GnRH antagonist protocol, the patients with fewer than 5 good quality embryos on day 3, and the patients receiving DET or SBT. The criteria of fewer than 5 good quality on day 3 derived from an RCT which indicated that patients might benefit from blastocyst culture with a threshold of four good embryos [24].

During the study period, the assignment of DET or SBT occurred on Day 3 based on a pre-ET consult with the knowledge of embryo quality and availability. DET was performed in 1,811 cycles and SBT was performed in 305 cycles. Propensity score (PS) matching was performed to reduce the bias between study groups (DET versus SBT transfer) resulting from certain baseline demographic, clinical, and embryologic confounding factors. After PS matching, there were 303 ET cycles in each group.

### Ovarian stimulation and oocyte retrieval

All patients received antagonist stimulation protocols. The starting dose of gonadotropin was adjusted according to antral follicle count (AFC), body mass index (BMI), the patient’s age, and follicular growth response. All patients received recombinant follicle-stimulating hormone (FSH) (Gonal-F®; Merck Serono) or hMG (Menotropins; Lizhu, China) stimulation in doses ranging from 150 to 300 IU, daily for up to 10 days. A GnRH antagonist (Cetrotide®; Merck Serono) was introduced when a leading follicle achieved 12 mm or the level of LH up to 3IU/L. Final oocyte maturation was induced with 250 µg of recombinant human chorionic gonadotropin (hCG) (Ovidrel®; Merck Serono) or 10000IU of HCG(Chorionic Gonadotropin; Lizhu, China) when at least one follicle reached a diameter of 18 mm or two follicles reached a diameter of 17 mm. All patients submitted to fresh embryo transfer. During COS, hormone measurements (LH, E2, and P4) were performed on the trigger day. Oocyte retrieval was performed 35–36 h after the trigger, guided by transvaginal ultrasound with a COOK 17-gauge oocyte recovery set (K-OPSD-1735-B-L; WilliamA. Cook Australia).

### Luteal support

The luteal phase was supported by one of two regimens: [1] vaginal micronized progesterone(Crinone® 8%; Merck Serono) beginning the day of oocyte retrieval, administered vaginally in a dose of 90 mg/d; [2] Intramuscular progesterone(Progesterone; Xianju, China) at the dosage of 40 mg/d from the day of retrieval. Progesterone was used for at least 14 days when a pregnancy test was performed, and until 10 weeks if the pregnancy was confirmed.

### Laboratory procedures

Following ovum pick-up (OPU), oocytes were inseminated using either conventional IVF or intracytoplasmic sperm injection (ICSI), with the fertilization results checked 18–20 h following insemination.

Embryos and blastocysts were all cultured in single droplets following insemination. All of the oocytes and embryos were cultured in Cook series media (KSIFM, KSICM, or KSIBM, Cook Medical, Bloomington, IN) with oil overlay (OVOIL, Vitrolife, Göteborg, Sweden) in traditional incubators (C200, Labotect, Göttingen, Germany) at 37 °C, 6% CO2, and 5% O2. Morphological evaluations were performed as previously described [25]. For cleavage-stage embryos, embryo quality was assessed with a combination of cell number, blastomere size, and degree of fragmentation. Grade I embryos (good quality) were defined as 8-cell embryos with evenly sized blastomeres and no more than 5% fragmentation. Grade II embryos (fair quality) were 7–10 cell embryos with minor defects in blastomere size or moderate fragmentation (10-15%). Grade III embryos (poor quality) were embryos with two of the following defects: cell number < 7 or > 10; abnormally sized blastomeres and a fragmentation rate of 10%. Embryos with severe fragmentation (> 50%) and embryos with a combination of more than two major defects were not considered for transfer.

The evaluation of blastocysts used the Gardner grading system [26]. Blastocysts scored as AA, AB, and BA were considered good quality. Blastocysts scored as BB were considered fair quality. Blastocysts scored as BC, CB, AC, and CA were considered poor quality. The blastocysts scored as CC were not considered for transfer. Only expanded blastocysts on Day 5 morning were considered for fresh transfer. The delayed blastocysts were left for extended culture and additional morphological evaluation was carried out on Days 6 and 7. As soon as the delayed blastocysts reached transferable criteria, they were cryopreserved for subsequent transfer.

Embryo transfers were performed by using a Cook catheter (K-JETS-7019-SIVF, Cook, IN, USA) under the guidance of abdominal ultrasonography. Assisted hatching was not performed during the period of study.

Following the fresh transfer, the surplus embryos or delayed blastocysts were cryopreserved with a vitrification protocol, employing 15% dimethyl sulfoxide, 15% ethylene glycol, and 0.6 M sucrose as cryoprotectants (K-SIBV, Cook) and a Cook Warming Kit (K-SIBW, Cook) was used for thawing. Blastocoelic volume was reduced before cryopreservation using a laser system (SATURN, RI, Falmouth, UK). Cryopreserved cleavage-stage embryos were not considered for blastocyst culture in our clinic.

## Outcomes

### Statistical analysis

The R packages ‘MatchIt’ and ‘outmatch’ were used to apply optimal matching in a 1:1 ratio [27]. The matchit function of the R package ‘MatchIt’ was applied to estimate the PS using logistic regression (logit) based on the following variables: women’s and men’s age at the start cycle, parity ≥ 1, gravidity, with or not endometriosis, with or not tubal factors, with or not PCOS, Female body mass index(BMI), basal FSH, basal LH, AFC, Estradiol(E2)on HCG day, progesterone(P) on HCG day, the thickness of endometrium on HCG day, the starting gonadotropin(Gn) for controlled ovarian stimulation(COS), oocyte yield, number of available embryos, the number of good/fair quality embryos on day 3, and insemination protocol.

PSs were compared using density plots. The baseline characteristics, ovarian stimulation outcomes, and clinical outcomes were evaluated before and after matching. The matched dataset was used to compare primary and secondary outcomes.

After PS-matching, We carried out four subgroup analyses for outcomes between the DET and SBT groups to test and verify a more appropriate embryo transfer strategy for different criteria of low day 3 embryo yield. Subgroups were carried out in the patients with fewer than 5 available embryos on day 3, the patients with fewer than 4 available embryos on day 3, the patients with fewer than 3 available embryos on day 3, and the patients with less than 4 oocytes retrieved.

Generalized linear models were used to adjust for potential residual confounding after matching.

Descriptive statistics were expressed as mean SD and median [IQR] for continuous data according to the distribution. Wilcoxon tests were used to compare two sets of continuous data.

Frequencies and percentages were used to present categorical data and were compared by chi-square tests. A *P*-value of < 0.05 was considered statistically significant.

Statistical analysis was performed using R version 4.1.

## Results

### Demographic and baseline IVF characteristics before and after PS matching

The demographic and baseline cycle characteristics, ovarian stimulation data, and the laboratory parameters of the study groups before/ after PS matching are shown in Table 1. The women’s and men’s age, parity ≥ 1, gravidity, and the infertility factors of endometriosis, tubal and PCOS or not, Female BMI, the level of basic FSH and LH, the number of AFC were comparable between the DET group and SBT group. The number of transfer cycles is a significant difference between the two groups(*P* < 0.001), and the first transfer cycles in the DET group were more than the SBT group before PS matching. After PS matching, the number of transfer cycles was comparable between the two groups(*P* = 0.805).

**Table 1:** Demographic and baseline IVF characteristics for PS matching

	Before PS matching				After PS matching		
	DET	SBT	*P*-value		DET	SBT	*P*-value
	(*N* = 1811)	(*N* = 305)			(*N* = 303)	(*N* = 303)	
**Female age, yr**							
Mean(SD)	34.7(4.90)	34.5(4.37)	0.345		34.2(4.75)	34.5(4.36)	0.568
**Male age, yr**							
Mean(SD)	36.3(5.69)	35.8(5.34)	0.15		35.3(5.42)	35.8(5.33)	0.256
**Parity ≥ 1**	583 (32.2%)	102 (33.4%)	0.715		102 (33.7%)	101 (33.3%)	1
**Gravidity**							
0	646 (35.7%)	91 (29.8%)	0.262		89 (29.4%)	91 (30.0%)	0.787
1	511 (28.2%)	95 (31.1%)			92 (30.4%)	93 (30.7%)	
2	460 (25.4%)	85 (27.9%)			94 (31.0%)	85 (28.1%)	
≥ 3	194 (10.7%)	34 (11.1%)			28 (9.2%)	34 (11.2%)	
**Number of transfer cycles**							
1	1241 (68.5%)	180 (59.0%)	< 0.001		172 (56.8%)	180 (59.4%)	0.805
2	347 (19.2%)	43 (14.1%)			46 (15.2%)	43 (14.2%)	
3	223 (12.3%)	82 (26.9%)			85 (28.1%)	80 (26.4%)	
Endometriosis	130 (7.2%)	24 (7.9%)	0.756		22 (7.3%)	24 (7.9%)	0.878
Tubal factors	1289 (71.2%)	219 (71.8%)	0.876		202 (66.7%)	218 (71.9%)	0.186
PCOS	15 (0.8%)	6 (2.0%)	0.123		7 (2.3%)	5 (1.7%)	0.771
**Female body mass index(BMI), kg/m2**							
Mean(SD)	21.5(2.49)	21.1(2.64)	0.0409		21.1(2.66)	21.1(2.64)	0.883
**Basal FSH, IU/L**							
Mean(SD)	8.70(3.35)	9.29(5.22)	0.0536		9.34(3.74)	9.08(3.89)	0.343
**Basal LH, IU/L**							
Mean(SD)	4.23(2.18)	4.28(2.56)	0.886		4.35(2.20)	4.25(2.47)	0.591
**AFC**							
Median [Q1,Q3]	6.00 [4.00,8.00]	6.00 [5.00,9.00]	0.115		6.00 [4.00,9.00]	6.00 [5.00,9.00]	0.931
**Estradiol(E2)on HCG day**							
Median [Q1,Q3]	1430 [999,2120]	1690 [1110,2610]	< 0.001		1630 [1010,2440]	1690 [1120,2600]	0.199
**Progesterone(P)on HCG day**							
Median [Q1,Q3]	0.820 [0.580,1.16]	0.860 [0.590,1.17]	0.442		0.790 [0.530,1.15]	0.870 [0.595,1.17]	0.105
**Endometrial thickness on HCG day, mm**							
Mean(SD)	10.0(2.30)	9.87(2.36)	0.358		9.95(2.25)	9.86(2.36)	0.681
**Starting dose of ovarian stimulation, IU**							
Median [Q1,Q3]	225 [225,225]	225 [225,225]	0.0633		225 [225,225]	225 [225,225]	0.209
**Oocyte yield**							
Median [Q1,Q3]	5.00 [3.00,7.00]	5.00 [3.00,7.00]	0.317		5.00 [3.00,6.00]	5.00 [3.00,7.00]	0.219
**Number of available embryos on day 3**							
Median [Q1,Q3]	3.00 [2.00,4.00]	3.00 [2.00,5.00]	0.0749		3.00 [2.00,4.00]	3.00 [2.00,5.00]	0.0217
**Number of good/fair quality embryos on day 3**							
Median [Q1,Q3]	2.00 [1.00,3.00]	2.00 [1.00,3.00]	< 0.001		2.00 [1.00,3.00]	2.00 [1.00,3.00]	0.0127
**Insemination protocol**							
ICSI	418 (23.1%)	56 (18.4%)	0.0265		59 (19.5%)	56 (18.5%)	0.915
IVF	1312 (72.4%)	242 (79.3%)			236 (77.9%)	240 (79.2%)	
RICSI	81 (4.5%)	7 (2.3%)			8 (2.6%)	7 (2.3%)	

The data of COS such as the level of Estradiol(E2)on HCG day was significantly lower in the DET group before PS matching (*P* < 0.001)and was comparable after PS matching (*P* = 0.199).

The number of available embryos on day 3 was no different before PS matching, but it was slightly more in the SBT goup than in the DET group after PS matching(*P* = 0.0217). The type of ART was slightly different between the two groups before PS matching(*P* = 0.0265) and was similar after PS matching(*P* = 0.915).

After PS matching, the number of good/fair quality embryos on day 3 was significantly lower in the SBT group (*P* = 0.0127). The percentage of cancellation of fresh transfer, freeze-all cycles, and no embryo transfer cycles was significantly higher in the SBT group than in the DET group (*P* < 0.001). There were 78 cycles in the SBT group and 0 cycles in the DET group canceled in fresh cycles. 78 cancellation cycles of fresh transfer in the SBT group included 34 no blastocysts formation on day 5 with consequent blastocysts on day 6 to freeze and 44 cycles completely no blastocysts formation. The median number of surplus frozen embryos was 0 embryos in the DET group, 1 embryo in the SBT group and the mean SD was 0.822 in the DET group, and 1.45 in the SBT group after PS matching (*P* < 0.001).

### Clinical outcomes

After PS matching, the percentage of clinical pregnancy and live births per OPU were significantly higher in the DET group (Table 2). The multiple pregnancy rate was significantly higher in the DET group after PS matching. The ectopic pregnancy rate did not differ between DET and SBT groups before PS matching(4.3% versus 0%, *P* = 0.0902) and after PS matching(3.3% versus 0%, *P* = 0.215) (Table 2).

**Table 2:** The laboratory parameters and clinic outcomes in fresh transfer cycle

	Before PS matching				After PS matching		
	DET		SBT	*P*-value	DET	SBT	*P*-value
	(*N* = 1811)		(*N* = 305)		(*N* = 303)	(*N* = 303)	
**2PN fertilization rate**							
Mean (SD)	73.1 (20.9)		69.8 (26.5)	0.301	72.5(22.2)	69.8(26.5)	0.544
**fertilization rate**							
Mean (SD)	85.1 (17.5)		87.8 (16.4)	0.0124	85.2(18.7)	87.8(16.4)	0.156
**Cancel fresh transfer**	0 (0%)		79 (25.9%)	< 0.001	**0 (0%)**	78 (25.7%)	< 0.001
**Freeze_all**	0 (0%)		34 (11.1%)	< 0.001	**0 (0%)**	34 (11.2%)	< 0.001
**No_embryo transfer**	0 (0%)		45 (14.8%)	< 0.001	0 (0%)	44 (14.5%)	< 0.001
**Number of surplus frozen embryos**							
Median [Q1,Q3]	1.00 [0,2.00]		1.00 [0,2.00]	< 0.001	0 [0,1.00]	1.00 [0,2.00]	< 0.001
**Embryo 1 Quality for transfer**							
Fair	1459 (80.6%)	140 (61.9%)		< 0.001	254 (83.8%)	139 (61.7%)	< 0.001
Good	154 (8.5%)		63 (27.9%)		24 (7.9%)	63 (28%)	
Poor	198 (10.9%)		23 (10.2%)		25 (8.3%)	23 (10.2%)	
**Embryo 2 Quality for transfer**							
Fair	1125 (62.1%)		-	-	190 (62.7%)	-	-
Good	39 (2.2%)		-		6 (2.0%)	-	
Poor	647 (35.7%)		-		107 (35.3%)	-	
**Clinical pregnancy per fresh transfer**	828 (45.7%)		87 (28.5%)	< 0.001	152 (50.2%)	87 (28.7%)	< 0.001
**Live birth per fresh transfer**	656 (36.2%)		65 (21.3%)	< 0.001	125 (41.3%)	65 (21.5%)	< 0.001
**Multiple pregnancies per fresh pregnancy**	241 (29.1%)		0 (0%)	< 0.001	45 (29.6%)	0 (0%)	< 0.001
**Ectopic pregnancy per fresh pregnancy**	36 (4.3%)		0 (0%)	0.0902	5 (3.3%)	0 (0%)	0.215

**Table 3:** The cumulative outcomes of per OPU

	Before PS matching			After PS matching		
	DET	SBT	*P*-value	DET	SBT	*P*-value
	(*N* = 1811)	(*N* = 305)		(*N* = 303)	(*N* = 305)	
**Cumulative clinical pregnancy**	957 (52.8%)	153 (50.2%)	0.421	177 (58.4%)	152 (50.2%)	0.0503
**Cumulative live birth(CLB)**	774 (42.7%)	123 (40.3%)	0.468	148 (48.8%)	122 (40.3%)	0.041
**Cumulative multiple pregnancies**	720 (39.8%)	24 (7.9%)	< 0.001	134 (44.2%)	24 (7.9%)	< 0.001
**Number of failed ET attempts**						
Median [Q1,Q3]	1.00 [0,2.00]	1.00 [0,2.00]	0.946	1.00 [0,2.00]	1.00 [0,2.00]	0.166
**Number of ET**						
Median [Q1,Q3]	1.00 [1.00,2.00]	1.00 [1.00,2.00]	0.851	1.00 [1.00,2.00]	1.00 [1.00,2.00]	0.341

### Cumulative outcomes

Marginal differences were observed for the CLBR per OPU after PS-matching(*P* = 0.041). The cumulative multiple pregnancy rate was significantly higher in the DET group after PS matching ( *P* < 0.001). The number of failed ET attempts (*P* = 0.166)and the number of ET per OPU, *P* = 0.341; Table 3 were no differences in the two groups, but the number of failed ET attempts and the number of ET to achieve live birth in the SBT group were more than in DET group (Table 4). Concerning the birth weight and gestational age, the DET birth cohort as a whole has a lower birth weight compared with the SBT cohort. However, when the data were split into singletons and twins, the differences were no longer significant (Table 5).

**Table 4:** The number of failed ET attempt or ET the achieve live birth

	Before PS matching			After PS matching		
	DET	SBT	*P*-value	DET	SBT	*P*-value
	(*N* = 774)	(*N* = 123)		(*N* = 148)	(*N* = 122)	
**Number_of_failed_ET_attempt to achieve LB**						
Median [Min, Max]	0 [0, 4.00]	0 [0, 2.00]	< 0.001	0 [0, 4.00]	0 [0, 2.00]	< 0.001
**Number_of_ET to achieve LB**						
Median [Min, Max]	1.00 [1.00, 5.00]	1.00 [1.00, 3.00]	< 0.001	1.00 [1.00, 5.00]	1.00 [1.00, 3.00]	< 0.001
**Number of thaw-transfer cycles**						
Median [Min, Max]	0 [0, 4.00]	1.00 [0, 3.00]	0.001	0 [0, 4.00]	1.00 [0, 3.00]	< 0.001

**Table 5:** Neonatal outcomes

	Before PS matching			After PS matching		
	DET	SBT	*P*-value	DET	SBT	*P*-value
**All birth**						
**Birth weight, g**						
Mean (SD)	3060 (540)	3180 (530)	0.0186	3040(533)	3180(532)	0.0254
**Gestational age, week**						
Mean(SD)	38.4(1.84)	38.6(2.09)		38.3(2.02)	38.5(2.10)	0.162
**preterm**						
	110 (14.3%)	12 (9.8%)	0.226	20 (15.4%)	12 (9.8%)	0.257
**Singleton**						
**Birth weight, g**						
Mean(SD)	3230(456)	3210(513)	0.759	3240(423)	3210(514)	0.785
**Gestational age, week**						
Mean(SD)	38.9(1.45)	38.7(2.00)	0.438	39.0(1.46)	38.7(2.01)	0.322
**preterm**						
	40 (6.8%)	9 (7.7%)	0.887	5 (5.3%)	9 (7.8%)	0.665
**Sex of children**						
Male	327 (55.7%)	62 (53.0%)	0.662	63 (58.3%)	62 (53.4%)	0.548
Female	260 (44.3%)	55 (47.0%)		45 (41.7%)	54 (46.6%)	
**Twin**						
**Birth weight, g**						
Mean(SD)	2520(399)	2530(468)	0.583	2520(444)	2530(468)	0.719
**Gestational age, week**						
Mean(SD)	36.7(1.85)	36.1(2.53)	0.583	36.5(1.90)	36.1(2.53)	0.776
**preterm**						
	68 (37.4%)	3 (50.0%)	0.841	14 (41.2%)	3 (50.0%)	1
**Sex of children**						
Male	201(55.2%)	7(58.3%)	0.831	52(70.3%)	7(58.3%)	0.409
Female	163(44.8%)	5(41.7%)		22(29.7%)	5(41.7%)	

### Subgoups outcomes

The laboratory parameters outcomes, such as the percentage of cancellation of fresh transfer, freeze-all cycles, and no embryo transfer cycles were similar to the main study(*P* < 0.001). Though the number of available embryos and good/fair quality embryos on day 3 in the SBT group was less than in the DET group in every subgroup, the comparison of the number of available embryos on day 3 in the subgroups with fewer than 5 available embryos was no statistical significance and were obviously significance in other subgroups and only in the patients with fewer than 3 available embryos the number of good/fair quality embryos on day 3 had obvious difference. The comparison of the number of surplus frozen embryos and the quality of embryos in the fresh transfer cycle was different in every subgroup.

The outcomes of CLBR, CMPR, LBR, CPR, and MPR in every subgroup were similar to the outcome of the main study. The CLBR was almost equal between the DET and SBT groups, the CMPR was significantly lower in the SBT group. The LBR, CPR, and MPR were higher in the DET group. The number of failed ET attempts and the number of ET to achieve live birth per OPU in all subgroups were significantly higher in the SBT group than in the DET group.

## Discussion

In this study, we compared DET versus SBT policy in patients with fewer than 5 good quality day 3 embryos matched according to relevant demographic, clinical, and embryologic characteristics. The CPR and LBR per fresh transfer were higher in the DET group, but only marginal differences were observed in the CLBR per OPU taking into consideration subsequent cryopreservation. However, the proportion of patients having fresh transfer cancellation, freeze-all cycles, and no embryo transfer cycles was significantly higher in SBT, which was possibly related to the delay or failure of blastocyst development. In addition, for those who finally achieved a live birth following the OPU, the number of failed ET attempts and the total number of ET per OPU was significantly higher in the SBT group, suggesting it took the patients a longer time to achieve a live birth with the SBT strategy. On the other hand, the MPR was still higher in the DET group. Taken together, the data suggested a trade-off between efficiency and safety: the SBT minimized the chance of multiple pregnancies with the cost of efficiency including cycle cancelation and more attempts to achieve a live birth. The additional subgroup analyses suggested that similar results could be expected in patients with fewer embryos or oocytes.

The comparative studies between blastocyst transfer and cleavage-stage embryo transfer have been investigated and debated for decades. However, the most recent Cochrane review still suggested that there is limited evidence to evaluate the performance of both strategies concerning cumulative outcomes following OPU, warranting future efforts on this issue [7]. Since the evaluation of the effectiveness of ART treatment has moved from the outcomes following a single transfer to the cumulative outcomes following multiple transfer attempts from the treatment, the number of attempts required to achieve a live birth could be a measure of efficiency [28]. Our study highlighted a potential difference between DET and SBT in terms of the number of attempts.

The number of gametes and embryos was a significant consideration when evaluating the patients` cumulative outcomes and the concerns for the failure of blastocyst formation may increase with a decreased embryo number. The early study suggested a threshold of four good embryos on day 3 to indicate that the patient would benefit from blastocyst transfer [24]. It was confirmed by a more recent study which suggested that blastocyst transfer is superior to cleavage transfer in terms of cumulative pregnancy rate when at least 4 zygotes are obtained [29]. Controversially, Yang et al.reported a similar ongoing pregnancy rate (25.84% versus 26.92%; odds ratio [OR] 0.95; 95% confidence interval [CI] 0.61–1.50) between the cleavage stage transfer (*n* = 267) and blastocyst transfer (*n* = 156) group in patients with 1–3 embryos available on day 3, even a cancellation rate of 24.36% were taken into account [19].

However, the blastocyst transfer group still has a higher number of day 3 embryos in that study. Croo et al.investigated a cohort of patients who had four or fewer zygotes on Day 1 and matched for a series of covariates including the number and quality of the embryos on Day 3, showing no difference in cumulative live birth rates between cleavage versus blastocyst transfer [30]. Nevertheless, the study also showed that 201 out of 571 blastocyst transfer cycles did not result in a blastocyst for subsequent transfer. It appeared that due to the relatively low reproductive efficiency of the poor prognosis patients, the effect of blastocyst formation failure on the cumulative outcomes was minimal. Our subgroup analyses also showed that the difference in cumulative birth rate diminished with decreasing number of gametes. However, the difference in the number of attempts to achieve a live birth remained significant.

With a given oocyte yield, cleavage stage culture is associated with greater numbers of embryos available, and blastocyst transfer is associated with increased risk with no embryos to transfer [31]. As the “normal” blastocyst development rate suggested by the Vienna consensus ranges from 40 to 60%, each of two viable cleavage stage embryos is expected to result in one blastocyst [32]. Assuming the same uterine environment, DET might require a similar number of day 3 embryos than SBT and therefore contribute similarly to the cumulative outcomes. However, the hypothesis that the uterine environment may provide essential substances that could not be obtained in the culture medium for arrested embryos may support the value of DET in patients with few viable embryos on day 3 [23]. The concept may be supported by the observation of patients with only one embryo available, where immediate transfer of day 3 embryo is superior to the attempt of extended culture in terms of live birth rate [20]. The retrospective study of Long et al. also showed that double cleavage-stage embryos led to a higher live birth rate, accompanied by increased risks of miscarriages, maternal complications, twin births, preterm births, and low birth weight [33].On the other hand, a recent study suggested that the number of day 3 embryos may not only affect the blastocyst availability of subsequent blastocyst transfer but also the blastocyst implantation. Patients with fewer than 4 good-quality day 3 embryos may suffer an unfavorable outcome following blastocyst transfer in comparison with those with 4 good-quality day 3 embryos or more [34].

Despite the slightly higher cumulative live birth rate and fewer failed attempts to achieve a live birth, our data also suggested that multiple pregnancies remained a considerable issue in the DET group. The multiple pregnancy rate did not decrease within the subgroups of fewer zygotes. Multiple pregnancies are associated with increased maternal, neonatal, and childhood morbidity and mortality and medical expenses either from individuals or society. However, the decision of embryo transfer strategy is never single-factor driven. There are many other factors beyond the goal of achieving a healthy singleton that can affect the clinical decision, such as age, prognosis, patient desires, and economic considerations [35]. Additionally, it is believed that not all multiple pregnancies turn out badly and not all singleton births turn out well, leading people to pay more attention to the higher pregnancy rate per transfer cycle and the economic pressure on the number of ART attempts and so on [36]. Keeping patients informed may play a crucial role during the process.

For the patients and clinicians who prefer DET over single embryo transfer, the fear of prolonging the time to conception and adversely affecting live birth chances is a major concern that prevents them from choosing single embryo transfer [37]. The concern may raised from the lack of confidence of the currently used morphological-based embryo election. With the ongoing advancements in technologies, newer technologies such as time-lapse monitoring [38]and artificial intelligence-assisted embryo selection [39] are introduced into the ART workflow. With an enhanced ability to select the most competent embryos and predict the ET outcome, the balance of the trade-off may move further toward single transfer. Nevertheless, there are still many challenges before the newer technologies are ready to become routine practice. For instance, a recent meta-analysis suggested that the use of time-lapse monitoring does not improve the outcomes of ET [40], calling for further investigation on the use of this technique.

Our study only included antagonist cycles. The GnRH antagonist protocol is recommended over the GnRH agonist protocols given the comparable efficacy and higher safety in the general IVF/ICSI population [41]. However, the effect of GnRH antagonists on endometrium has undergone years of debate [42]. Changes in endometrial expression profiles in antagonist cycles compared with agonist and natural cycles have been found, suggesting a potential alternation in the implantation window [43]. The histological study may suggest an advanced endometrial maturation correlating with altered gene expression in GnRH antagonist cycles [44]. Patients with advanced endometrium might miss the window of implantation if an extended culture is scheduled. It is also supported by the study showing that a freeze-all strategy following GnRH antagonist cycles may improve implantation, clinical pregnancy rate, and ongoing pregnancy rate [45]. It might bias the conclusion drawn from the study.

### Limitations

Our study is limited by its retrospective design. Although various potential confounders and inconsistencies in treatments were matched and adjusted for, the bias of patient assignment still existed. For instance, a patient who chose to receive DET may be due to her desire to shorten the treatment and a patient receiving SBT may tend to prevent the risks of pregnancy complications. Unknown or unmeasured confounders might still affect the outcome of the study.

Another drawback of the study was that we failed to demonstrate a complete risk profile for both DET and SBT, due to the limitation of the dataset. The adverse effects of twin pregnancy affect both mothers and babies. Beyond the neonatal outcomes, complications such as postpartum hemorrhage, gestational diabetes, and hypertension should also be a significant concern and their risks should be carefully informed to the patients.

Caution should also be taken due to the population in the study is relatively young. It may limit the generalizability of the conclusion in older cohorts. The difference between DET and SBT may be narrowed in patients with poorer prognoses as the overall chance of getting LB is low, but the risk of multiple pregnancies remains significant (Supplementary Table 1). However, older patients might also have a lower blastocyst formation rate [46] and thus increase the cancellation.

## Conclusions

Our study suggested that despite a marginal difference in cumulative live birth rate the transfer strategies of SBT and DET also diverge in the trade-off of safety and efficiency. The selection of transfer strategy is not only efficacy-driven but also scenario-dependent. The patients with higher expectations of success rate may be less accepting of cycle cancellation or delayed transfer while patients with risk factors for pregnancy complications might be prone to choose a safer way.

### Electronic supplementary material

Below is the link to the electronic supplementary material.

Supplementary Material 1
